# Evaluating the Efficacy and Safety of Stereotactic Arrhythmia Radioablation in Ventricular Tachycardia: A Comprehensive Systematic Review and Meta-Analysis

**Published:** 2025-01-31

**Authors:** Keyur D. Shah, Chih-Wei Chang, Sibo Tian, Pretesh Patel, Richard Qiu, Justin Roper, Jun Zhou, Zhen Tian, Xiaofeng Yang

**Affiliations:** 1 Department of Radiation Oncology and Winship Cancer Institute, Emory University, Atlanta, GA; 2 Department of Radiation & Cellular Oncology, University of Chicago, Chicago, IL

**Keywords:** Stereotactic Arrhythmia Radioablation (STAR), Stereotactic Body Radiation Therapy (SBRT), Ventricular Tachycardia (VT), Cardiac Radiosurgery, Noninvasive VT Ablation

## Abstract

**Purpose:**

Stereotactic arrhythmia radioablation (STAR) has emerged as a promising non-invasive treatment for refractory ventricular tachycardia (VT), offering a novel alternative for patients who are poor candidates for catheter ablation. This systematic review and meta-analysis evaluates the safety, efficacy, and technical aspects of STAR across preclinical studies, case reports, case series, and clinical trials.

**Methods and Materials:**

A systematic review identified 80 studies published between 2015 and 2024, including 12 preclinical studies, 47 case reports, 15 case series, and 6 clinical trials. Data on patient demographics, treatment parameters, and clinical outcomes were extracted. Meta-analyses were performed for pooled mortality rates, VT burden reduction, and acute toxicities, with subgroup analyses exploring cardiomyopathy type, age, left ventricular ejection fraction (LVEF), and treatment modality.

**Results:**

The pooled 6- and 12-month mortality rates were 16% (95% CI: 11–21%) and 32% (95% CI: 26–39%), respectively. VT burden reduction at 6 months was 75% (95% CI: 73–77%), with significant heterogeneity (I^2^ = 98.8%). Grade 3+ acute toxicities were observed in 7% (95% CI: 4–11%), with pneumonitis being the most common. Subgroup analyses showed comparable outcomes between LINAC- and CyberKnife-based treatments, with minor differences based on patient characteristics and cardiomyopathy type.

**Conclusions:**

STAR demonstrates significant potential in reducing VT burden and improving patient outcomes. While favorable acute safety profiles and efficacy support clinical adoption, variability in treatment protocols underscores the need for standardized practices. Future studies should aim to optimize patient selection, establish robust dosimetric standards, and evaluate long-term safety.

## 1. Introduction

Ventricular tachycardia (VT) is a life-threatening arrhythmia characterized by rapid electrical activity originating in the ventricles, which can compromise cardiac output and lead to hemodynamic instability (1, 2). VT circuits are commonly associated with myocardial scar tissue, typically resulting from conditions such as myocardial infarction or cardiomyopathies (3, 4). The reentrant mechanism is a prevalent cause of VT, where electrical impulses circulate through areas of slow conduction, including the central isthmus, entrance, and exit sites, which sustain arrhythmic activity (5, 6). Between 2007 and 2020, ventricular tachycardia (VT) was linked to over 7,000 deaths in the United States among patients with underlying cardiovascular disease, underscoring the significant mortality burden associated with this arrhythmia (7).

The management of VT includes pharmacological therapy, device-based interventions, and catheter ablation (8). Antiarrhythmic drugs such as amiodarone and beta-blockers are first-line treatments, but their efficacy is often limited, and they may carry significant side effects (9). Implantable cardioverter-defibrillators (ICDs) are a cornerstone in preventing sudden cardiac death by delivering shocks to terminate VT episodes (10); however, they do not prevent arrhythmia recurrence and can negatively impact the quality of life due to frequent shocks and complications (11).

Catheter ablation has emerged as a primary interventional strategy, aiming to eliminate arrhythmogenic substrates by using radiofrequency or cryothermal energy to create lesions that disrupt reentrant circuits (12). Despite its effectiveness, catheter ablation is invasive and may be challenging in patients with extensive myocardial scar burden or hemodynamic instability. Procedural success is highly dependent on precise identification and targeting of VT circuits, often guided by electroanatomical mapping (EAM), which integrates functional and anatomical data for improved precision. While catheter ablation is a primary interventional strategy for VT, it is not curative in all cases, particularly in patients with extensive scar burden or inaccessible arrhythmogenic tissue. Additionally, the procedure is associated with elevated risks of complications and mortality, especially in those with advanced heart failure or significant comorbidities (13, 14).

Stereotactic arrhythmia radioablation (STAR) has emerged as a promising non-invasive alternative for treating VT, particularly in patients who are poor candidates for catheter ablation. STAR offers a non-invasive alternative for VT treatment, leveraging high-dose radiation to modify arrhythmogenic substrates. Unlike catheter-based interventions, STAR eliminates the risks of invasive procedures while providing precision targeting through advanced imaging modalities (15). The concept of using radiation therapy for VT management dates to the early 2000s in Japan. Miyashita et al (16) pioneered this approach by combining chemotherapy and RT to treat a case of right ventricular outflow tract (RVOT) VT in a 70-year-old female, delivering 40 Gy in a single fraction. This early effort was followed by Tanaka et al (17), who applied a similar chemo-RT regimen with 51 Gy in a single fraction to manage RVOT VT in a 65-year-old male. While these exploratory studies highlighted the potential of RT to address arrhythmic substrates, the lack of advanced imaging and delivery techniques limited the precision and safety of these early treatments.

The modern era of STAR began in 2015 when Loo et al (18) at Stanford demonstrated the first in-human STAR procedure as it is known today. Using the CyberKnife system, they delivered 25 Gy in a single fraction to treat VT and achieved a remarkable 90.75% reduction in arrhythmic events. This groundbreaking work established the feasibility of non-invasive VT ablation and paved the way for further exploration. In 2017, Cuculich et al (19) reported the first case series of STAR, treating five patients with 25 Gy in a single fraction and demonstrating an impressive 99.99% reduction in VT events. This study marked a pivotal moment in the field, showcasing STAR's clinical efficacy and safety.

Utilizing advanced imaging modalities and precision delivery platforms, STAR delivers highly conformal radiation doses to arrhythmogenic substrates. Its key advantages include non-invasive delivery, outpatient feasibility, and the ability to target arrhythmogenic foci inaccessible by catheter-based approaches. The growing body of evidence supports STAR as an effective option for reducing VT burden, with favorable acute and mid-term outcomes. Most STAR treatments prescribe a dose of 25 Gy in a single fraction, guided by preclinical studies and clinical experience, with delivery platforms such as linear accelerators (LINACs), CyberKnife, and MRI-guided systems offering unique capabilities in motion management and precision targeting.

Given the increasing adoption of STAR for VT management, there is a need for a comprehensive synthesis of the available evidence to assess its safety and efficacy. This systematic review and meta-analysis aims to:

- Quantify mortality rates at 6- and 12-months following STAR for VT.
- Assess the efficacy of STAR in reducing VT burden, including subgroup analyses based on delivery modality, patient characteristics, and cardiomyopathy type.
- Evaluate the incidence of acute grade 3+ toxicities associated with STAR.

This systematic review and meta-analysis will include data from preclinical studies, case reports, case series, and clinical trials, offering a comprehensive perspective on the evolving role of STAR in VT management. The findings will help guide clinical practice and future research endeavors to optimize patient selection, treatment planning, and post-treatment monitoring.

## 2. Methods

### 2.1 Search Strategy and Study Selection

A comprehensive literature search was conducted to identify relevant studies evaluating the use of radiation therapy for VT. The search was performed in PubMed, using the search term: "ventricular tachycardia AND radiation therapy." The search covered studies published up to January 25, 2025, with filters applied to include specific study designs such as preclinical investigations, case reports, case series, and clinical trials. A total of 341 studies were initially identified through the database search.

The Preferred Reporting Items for Systematic Reviews and Meta-Analyses (PRISMA) (20) guidelines were followed to ensure a systematic and transparent selection process. After the removal of duplicates, the remaining studies underwent screening based on title and abstract, followed by full-text review.

Inclusion criteria were as follows:

- Studies reporting on preclinical investigations, case reports, case series, and clinical trials.
- Studies published in English.
- Studies reporting on distinct patient populations; in cases where multiple studies reported on the same cohort, the most recent publication was included.

Studies were excluded if they:

- Insufficient reporting on VT treatment using radiation therapy.
- Studies focused on unrelated topics, lacking clinical or preclinical relevance to radiation therapy for VT.
- Review articles, editorials, or commentaries that did not report original data.

**Figure 1.**
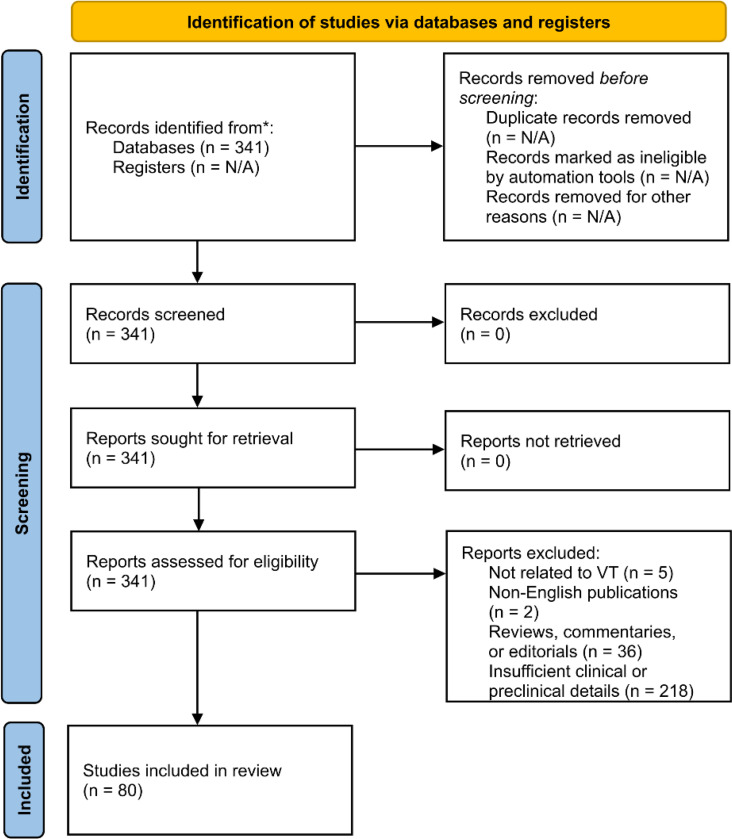
PRISMA Flow Diagram of Study Selection Process. Illustrates the systematic selection process for included studies, detailing the number of records identified, screened, excluded, and ultimately included in this systematic review and meta-analysis. Reasons for exclusion are categorized, including unrelated topics, insufficient data, duplicates, and non-English publications. Modified from Page et al (20).

Figure 1 illustrates the study selection process, detailing the number of records screened, included, and excluded at each stage.

### 2.2 Data Extraction and Analysis

Data were systematically extracted from the included studies, focusing on key parameters related to patient demographics, treatment characteristics, and clinical outcomes. The following variables were collected:

- Patient Characteristics: Sample size, median age, gender distribution, underlying cardiomyopathy (ischemic vs. non-ischemic), median left ventricular ejection fraction (LVEF) and prior catheter ablation history.
- Treatment Parameters: Radiation delivery modality (LINAC, CyberKnife, or MRI-guided systems), prescribed dose (typically 25 Gy in a single fraction), and planning target volume (PTV) margins.
- Clinical Outcomes: 6- and 12-month mortality rates, VT burden reduction, specifically %VT reduction at 6 months, adverse events (grade 3+ toxicity) within 90 days.

When data were missing or ambiguously reported, the studies were excluded from the meta-analysis for that specific variable to maintain the robustness of results.

### 2.3 Statistical Analysis

The meta-analysis was performed using a random-effects model to account for potential variability across the included studies. Key outcomes analyzed included mortality rates, efficacy measures, and safety profiles. Mortality rates were evaluated at 6 and 12 months, with subgroup analyses conducted to examine differences based on cardiomyopathy type (ischemic vs. non-ischemic), age (≤ median vs. > median), and LVEF (≤ median vs. > median). Treatment efficacy was assessed through pooled analysis of VT burden reduction, quantified as the percentage reduction in VT events at 6 months. Safety outcomes were reported as rates of grade 3 or higher adverse events occurring within 90 days of treatment.

Heterogeneity across studies was assessed using the I-squared (I^2^) statistic and Cochran’s Q test. I^2^ values of 50% or higher were interpreted as indicative of moderate-to-high heterogeneity, and potential sources of heterogeneity were further explored through subgroup analyses. These subgroup analyses included comparisons between LINAC- and CyberKnife-based treatments and evaluations of the impact of patient demographics, such as age and LVEF, on treatment outcomes. All statistical analyses were performed using Python, utilizing the Statsmodels and SciPy libraries. Results were presented as pooled estimates with corresponding 95% confidence intervals (CIs).

## 3. Results

### 3.1 Study Selection and Characteristics

A total of 341 studies were reviewed, and 80 studies were included in this systematic review and meta-analysis, comprising 12 preclinical studies, 47 case reports, 15 case series, and 6 clinical trials, published between 2015 and 2024. The PRISMA flow diagram summarizing the study selection process is presented in Figure 1.

Studies were conducted across 18 countries, with the majority originating from Europe (n = 45), followed by North America (n = 22) and Asia (n = 13). A total of 327 patients were analyzed across the included studies, with 87% male patients. Nearly 90% of the studies utilized 6 MV photons as the primary treatment modality, with 25 Gy prescribed as the standard dose to the arrhythmic substrate. The methods for defining target volumes varied across studies. However, the majority of studies incorporated cardiac computed tomography (CT) for anatomical localization and 12-lead electrocardiography (ECG) for arrhythmic mapping. Advanced techniques such as EAM or electrocardiographic imaging (ECGI) were frequently employed to refine the target definition and delineate arrhythmogenic substrates.

Variability was observed in the reporting of outcomes, with some studies focusing on VT burden reduction and others prioritizing survival and toxicity as primary endpoints. Given the heterogeneity in study designs and follow-up durations, pooled analyses with subgroup considerations (e.g., treatment modalities and motion management strategies) were conducted to provide a comprehensive assessment of outcomes.

### 3.2 Preclinical Studies

A total of 12 preclinical studies explored the effects of STAR across diverse animal models, including pigs (n = 81), rabbits (n = 32), dogs (n = 25), and rats (n = 9), representing a combined total of 173 animals. These studies spanned five countries (USA, Japan, Germany, Russia, and South Korea) and provided critical insights into the efficacy and safety of STAR. he key characteristics and findings from these studies are summarized in Table 1. Most studies assessed the impact of STAR on arrhythmia suppression, myocardial remodeling, conduction properties, and treatment safety. Photon-based STAR was the most commonly studied modality in preclinical studies (42%, n = 5), reflecting its widespread clinical adoption for arrhythmia management across various settings. Particle therapy modalities, including protons and carbon ions, were equally studied (33%, n = 4 each), highlighting growing interest in their precision for treating arrhythmogenic substrates. These studies assessed the impact of radiation on arrhythmia management, myocardial remodeling, and treatment safety. Amino et al. (21, 22) demonstrated dose-dependent reductions in VT/VF inducibility, with higher doses leading to improved conduction parameters. Lehmann et al. (23, 24) focused on achieving complete AV block with escalating doses, while Zei et al. (25) reported successful electrical isolation of the RSPV, emphasizing the impact of STAR on conduction pathways and arrhythmia suppression. Structural studies, such as those by Hohmann et al. (26, 27) and Kancharla et al. (28), highlighted enhanced scar homogenization and stabilization of cardiac function post-MI. Molecular analysis from Kim et al. (29) provided insights into early proteomic changes linked to radiation-induced stress responses.

While STAR demonstrated promising efficacy, safety concerns were noted in specific studies. Takami et al. (30) reported pericardial effusion in irradiated rabbits, while Imamura et al. (31) observed conduction slowing and structural remodeling in long-term follow-up. Studies such as Vaskovskii et al. (32) explored photon therapy's impact on AV node ablation, demonstrating dose-dependent conduction block effects. These findings emphasize the need for precise dose optimization.

### 3.3 Case Reports

The 47 included case reports, published between 2015 and 2024, provided detailed insights into individual patient experiences with STAR for recurrent VT. These studies predominantly utilized photon-based STAR, with 25 Gy in a single fraction being the standard dose prescription. Technologies used included LINAC systems, CyberKnife, and MRI-guided systems, with a variety of motion management strategies such as 4DCT, internal target volume (ITV) expansion, and fiducial marker-based tracking.

Most cases targeted monomorphic VT (MMVT), while polymorphic VT (PMVT) was less commonly reported. PTV volumes varied significantly across cases, reflecting differences in arrhythmogenic substrate sizes and target delineation strategies. Notably, motion management techniques were adapted based on the technology used, with CyberKnife treatments employing fiducial markers and LINAC systems relying on 4DCT and ITV expansion.

Outcomes from these reports highlighted the efficacy of STAR in reducing VT burden (96.73% ± 6.41%), often achieving substantial suppression of arrhythmic episodes. While acute toxicities were rare, a few patients experienced pneumonitis or exacerbation of chronic obstructive pulmonary disease (COPD). However, long-term follow-up data were inconsistently reported, limiting the ability to draw definitive conclusions about the incidence and severity of late effects. A detailed summary of individual case reports, including treatment characteristics, is provided in Table 2.

### 3.4 Clinical Series

A total of 21 clinical trials and case series were included, with sample sizes ranging from 3 to 36 patients. These studies provided valuable insights into the efficacy of STAR for recurrent ventricular tachycardia (VT) in larger cohorts. Across these studies, PTV values varied significantly, with a median of 81.55 cc (range: 14–330 cc), reflecting variability in target delineation practices and arrhythmogenic substrate sizes. Margins used for target volume expansion were inconsistent, ranging from 1 mm to 8 mm isotropic expansion, further emphasizing the variability in contouring practices across institutions.

The majority of studies (n = 19, 90.5%) employed photon-based STAR, with doses predominantly prescribed at 25 Gy in a single fraction. Of these, LINAC-based systems were used in 76.2% of cases (n = 16), while CyberKnife treatments were reported in 19.0% (n = 4). A small subset of studies used MRI-guided STAR systems (4.8%, n = 1), showcasing emerging technology for arrhythmia ablation.

Patient characteristics revealed significant baseline cardiac dysfunction, with a median LVEF of 27.5% (range: 10–72%). Ischemic cardiomyopathy (ICM) and non-ischemic cardiomyopathy (NICM) were nearly equally distributed, representing 51.81% and 48.19% of patients, respectively. Motion management techniques included 4DCT, ITV expansion, and fiducial marker-based tracking. 4DCT was the most frequently employed strategy, particularly in LINAC-based treatments, while fiducial tracking was utilized for CyberKnife treatments. However, details on motion management were inconsistently reported in some studies, limiting the ability to evaluate specific trends.

On average, a 75.6% reduction in VT burden was observed at six months, highlighting the substantial arrhythmic suppression achieved with STAR. Details of the included clinical trials and case series, including patient demographics, cardiomyopathy classification, and treatment characteristics, are summarized in Table 3.

### 3.5 Meta-Analysis

#### 3.5.1 6-Month and 12-Month Mortality

The pooled proportion of deaths at 6 months was 16% (95% CI: 11–21%), with minimal heterogeneity across studies (I^2^ = 0.00%, Cochran’s Q = 16.95, p = 0.56). For 12-month mortality, the pooled estimate was 32% (95% CI: 26–39%), also demonstrating low heterogeneity (I^2^ = 0.00%, Cochran’s Q = 15.45, p = 0.56). Certain studies, such as Haskova et al (90) contributed a high number of deaths at 12 months (e.g., 17/36), which could skew the overall pooled mortality rates at this time point. Such imbalances emphasize the importance of consistently reporting outcomes at standardized intervals, including short-term and intermediate time points, to allow accurate comparisons.

**Figure 2.**
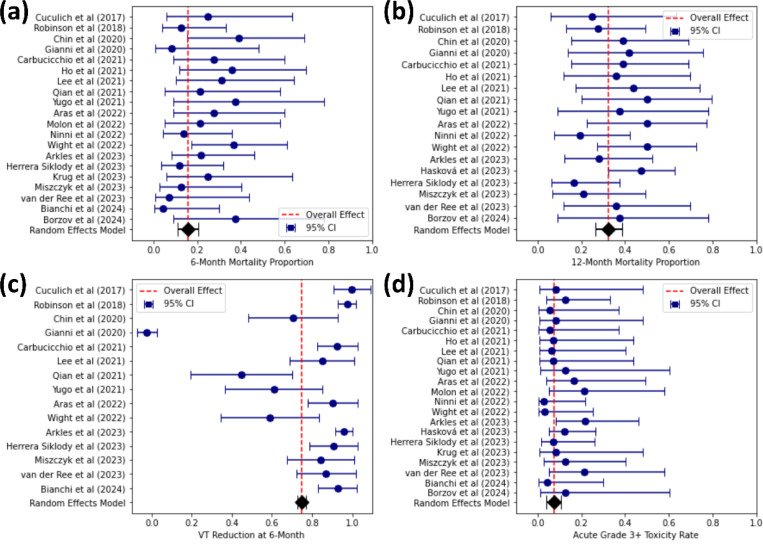
Forest Plots Summarizing Meta-Analysis Results for Mortality, VT Burden Reduction, and Acute Toxicity. Forest plots displaying the pooled effect estimates and 95% confidence intervals (CI) for **(a)** 6-month mortality, **(b)** 12-month mortality, **(c)** VT reduction at 6 months, and **(d)** acute grade 3+ toxicity rates within 90 days. The red dashed line represents the overall effect, while individual blue points and bars represent study-specific estimates and their CIs. A random-effects model was used for meta-analysis.

Figures 2(a) and 2(b) present the forest plots for 6-month and 12-month mortality, respectively.

#### 3.5.2 Grade 3+ Acute Toxicities

The pooled rate of grade 3+ adverse events within 90 days of treatment was 7% (95% CI: 4–11%), with no observed heterogeneity (I^2^ = 0.00%, Cochran’s Q = 8.6, p = 0.99). Toxicities included pneumonitis, exacerbation of chronic obstructive pulmonary disease (COPD), and gastrointestinal symptoms. The forest plot for acute toxicity rates is shown in Figure 2(c).

#### 3.5.3 VT Events Reduction at 6 Months

The pooled percentage reduction in VT events at 6 months was 75% (95% CI: 73–77%), with substantial heterogeneity (I^2^ = 98.80%, Cochran’s Q = 1163.39, p < 0.05). Figure 2(d) illustrates the forest plot for VT event reduction.

#### 3.5.4 Sub-group Analysis

Subgroup analyses provided additional insights into factors influencing outcomes. Comparisons between LINAC- and CyberKnife-based treatments revealed similar mortality rates at 12 months (35%, 95% CI: 27–43% for LINAC vs. 30%, 95% CI: 20–39% for CyberKnife), with minimal differences in acute toxicity (8%, 95% CI: 4–13% for LINAC vs. 6%, 95% CI: 1–12% for CyberKnife). Age-stratified analysis showed slightly lower mortality at 6 months for patients younger than the median age (14%, 95% CI: 9–19%) compared to older patients (22%, 95% CI: 11–33%). Similarly, patients with LVEF above the median had marginally lower mortality at 6 months (13%, 95% CI: 6–19%) compared to those with LVEF below the median (18%, 95% CI: 9–26%). Regarding cardiomyopathy types, mortality at 6 months was slightly lower for patients with NICM (13%, 95% CI: 6–20%) compared to ICM (16%, 95% CI: 9–23%), while VT burden reduction was higher for NICM group (99% for NICM vs. 59% for ICM). The analysis for VT burden reduction showed significant heterogeneity (I^2^ = 98.80%), indicating substantial variability across studies. This heterogeneity underscores the need for standardized reporting and consistent methodologies in future investigations. Factors such as differences in patient selection criteria, treatment protocols, and follow-up durations likely contributed to this variability. Despite the high heterogeneity, the overall pooled VT reduction of 75% (95% CI: 73–77%) demonstrates the promising efficacy of STAR in arrhythmia control across diverse settings.

These findings suggest that clinical outcomes of STAR for VT are generally consistent across subgroups, with minor differences that warrant further investigation, particularly for understanding variability in VT reduction outcomes.

## 4. Discussion

This systematic review and meta-analysis evaluated the safety, efficacy, and outcomes of stereotactic arrhythmia radioablation (STAR) for ventricular tachycardia (VT). Across included studies, pooled mortality rates at 6 and 12 months were 16% (95% CI: 11–21%) and 32% (95% CI: 26–39%), respectively, underscoring the high-risk profile of this patient population. Notably, one study (90)contributed disproportionately to the 12-month mortality estimate, reporting 17 of 36 deaths at 12 months but without data at 6 months. This variability highlights the need for standardized reporting practices, as differences in follow-up durations and incomplete data likely skew pooled estimates. A significant VT burden reduction of 75% (95% CI: 73–77%) at 6 months further demonstrated the promise of STAR in suppressing arrhythmic episodes.

Subgroup analyses revealed important insights into factors influencing outcomes. Younger patients and those with higher LVEF consistently demonstrated better survival and VT reduction rates. As compared to patients with ICM, NICM patients experienced superior VT burden reduction (99% vs. 59%) and reduced mortality at 6 months (16% vs 13%). These findings suggest that STAR outcomes may vary based on patient-specific characteristics, highlighting the need for tailored treatment approaches and stratified clinical trial designs.

The high heterogeneity observed in VT burden reduction outcomes (I^2^ = 98.80%) highlights a critical need for standardized reporting and consistent methodologies. Definitions of VT burden varied across studies, with some quantifying episodes per unit time and others measuring total VT events. Such variability complicates comparisons and meta-analyses, emphasizing the importance of establishing reporting frameworks akin to TRIPOD (94) standards for AI studies. Subgroup analyses identified younger patients, NICM, and higher LVEF as predictors of favorable outcomes. Future clinical trials should stratify patients based on these factors and consider incorporating interim analyses or predefined endpoints, such as VT-free survival, to evaluate efficacy or safety. Trials may also include provisions for early conclusion if predefined thresholds for success or excessive adverse events are met, ensuring patient safety and resource optimization. This approach would not only improve trial efficiency but also minimize risk for high-risk patients. The results also highlight the need to explore differential outcomes between LINAC- and CyberKnife-based treatments, particularly in terms of toxicity profiles and cost-effectiveness.

Accurate motion management remains a cornerstone of STAR. LINAC-based systems predominantly rely on 4DCT and ITV expansions, while CyberKnife employs fiducial tracking to accommodate respiratory and cardiac motion. Although CyberKnife offers sub-millimeter precision, its treatment times are significantly longer compared to LINACs (120 minutes vs 30 minutes), posing logistical challenges in a clinical setting. The variability in PTV margins across studies (ranging from 1 mm to 8 mm isotropic expansions) further underscores the lack of standardization in STAR planning. Addressing these inconsistencies is critical for optimizing treatment precision and minimizing radiation dose to surrounding organs.

Particle therapies, such as protons and carbon ions, represent an emerging frontier in STAR, particularly for younger patients or those with complex anatomies. The ability to leverage the Bragg peak for precise dose deposition makes these modalities uniquely suited for cases involving critical adjacent structures, such as the esophagus and lungs. Preclinical studies have demonstrated the feasibility of particle therapy for arrhythmia ablation (30, 93, 31); however, clinical data remain sparse. Dusi et al (43) and Amino et al (71) successfully demonstrated the first-in-human use of protons and carbons to treat VT and demonstrated a reduction in VT events post STAR.

Lee et al (47) treated an 11-year-old pediatric patient for VT with photons. While the patient was in good condition at the 3-month follow-up visit, long-term follow-up data are unavailable, and this patient might have benefited from proton therapy, given its dosimetric advantages. Shah et al (95) demonstrated significant reductions in OAR doses for retrospectively planned patients treated with proton therapy compared with corresponding photon plans. Despite these promising developments, challenges such as range uncertainties, motion management, and the high costs of particle therapy must be addressed to facilitate its broader adoption.

Grade 3+ adverse events were rare (7%, 95% CI: 4–11%) but underscore the importance of meticulous planning to avoid significant complications. The reported cases of esophagitis and pneumonitis, particularly in patients with posterior substrates, emphasize the need for advanced planning techniques and possibly proton therapy to spare critical structures. Moreover, attention must be paid to the radiation dose delivered to implantable cardioverter-defibrillators (ICDs), as inappropriate shocks or device malfunctions remain a concern. Guidelines such as AAPM TG-203 (96) offer practical recommendations for managing these challenges during treatment.

Cases requiring repeated STAR treatments illustrate the challenges of achieving complete arrhythmic suppression in patients with extensive or complex arrhythmogenic substrates. Improved target identification, supported by EAM, ECGI, and advanced imaging modalities, can mitigate the need for retreatments. Artificial intelligence-based automated models and semi-automated have shown promise in automating substrate delineation, reducing inter-observer variability, and identifying non-responders earlier in the treatment course (97–100).

The efficacy of STAR is underpinned by its ability to induce fibrosis and alter myocardial conduction properties, disrupting arrhythmogenic circuits. However, the biological mechanisms remain incompletely understood. Preclinical studies have highlighted changes in gap junction remodeling (e.g., connexin-43 expression), conduction slowing, and fibrosis as key contributors to arrhythmia suppression (21, 22). Long-term consequences of radiation, including vascular damage and inflammatory responses, require further investigation, particularly in younger patients who may face increased risks of late toxicities.

The overwhelming majority of STAR studies have been conducted in North America, Europe, and East Asia, with limited representation from South America, Africa, the Middle East, and the Indian subcontinent. This geographic disparity reflects broader inequities in access to advanced radiation therapy technologies. Efforts must be made to globalize radiation therapy, making STAR accessible to all patients who need it. This includes reducing financial and logistical barriers to acquiring treatment infrastructure and fostering international collaboration to ensure equitable access.

This study has several limitations: first, the high heterogeneity across studies, particularly in VT burden reduction outcomes, reflects inconsistencies in patient selection, treatment protocols, and reporting standards. Second, the exclusion of non-English studies may have introduced bias by omitting data from regions with significant STAR experience. Third, limited long-term follow-up in most studies precludes a comprehensive assessment of late toxicities and long-term arrhythmic recurrence. Lastly, the scarcity of randomized controlled trials limits the generalizability of findings.

Standardization of STAR protocols and reporting practices is critical to advancing this field. Collaborative efforts are needed to develop robust frameworks for patient selection, target delineation, and outcome reporting. Future research should focus on:

1. Expanding STAR to earlier-stage VT patients, including those without prior catheter ablation failure.
2. Exploring particle therapy, particularly protons, for cases requiring enhanced OAR sparing.
3. Investigating advanced imaging and motion management techniques to improve precision.
4. Addressing geographic disparities by fostering international collaborations and improving access to radiation therapy technologies in underserved regions.

The findings of this systematic review and meta-analysis underscore the transformative potential of STAR in VT management, while highlighting opportunities to refine and expand its application. Addressing the outlined challenges will be critical to maximizing STAR's clinical impact and ensuring equitable access to this life-saving technology.

In conclusion, STAR is poised to reshape VT management, bridging gaps in treatment options for patients unfit for catheter ablation. With continued collaboration, technological advancements, and equitable implementation, STAR holds the promise of evolving from an innovative alternative to an essential pillar of arrhythmia care.

## Figures and Tables

**Table 1.** Summary of Preclinical Studies Investigating Stereotactic Arrhythmia Radioablation (STAR) and Particle Therapy for Cardiac Applications.

Authors (Year)	Country	Animal (n)	Disease Model	Modality (Dose Gy)	Key Findings
Lehmann et al (2015)(23)	USA/Germany	Pigs (4)	Explanted	Carbons (70/90/160 Gy)	No AV block up to 130 Gy; complete AV block at 160 Gy, confirmed by PET-CT; no visible myocardial damage.
Amino et al (2017) (21)	Japan	Dogs (8)	AF	Carbons (15 Gy)	VT/VF inducibility reduced (25% vs. 100%) (irradiated (n=4) vs non-irradiated (n=4)); improved conduction (QRS & RMS40); increased C×43 (24–45%).
Lehmann et al (2017) (24)	USA	Pigs (10)	AV Junction Ablation	Photons (25/40/50/55 Gy)	Complete AV block achieved in 6/7 irradiated pigs (86%); lesion size increased with dose; no short-term side effects; no damage to esophagus, phrenic nerves, or trachea; histology revealed beam effects outside target volume.
Zei et al (2018) (25)	USA	Dogs + Pigs (19)	VT	Photons (15/20/25/35 Gy)	Successful electrical isolation of the RSPV achieved at 25 and 35 Gy (100%), partial isolation at 20 Gy (80%) and 15 Gy (50%); no complications or collateral tissue injury; transmural scar formation confirmed by histopathology.
Hohmann et al (2019) (26)	USA	Pigs (20)	LV Ablation (Healthy)	Protons (30/40 Gy)	Dose-dependent decline in LVEF (r = - 0.69, P = .008); LV dilation correlated with dose (r = 0.75, P = .003); functional decline observed ~3 months post-treatment.
Hohmann et al (2020) (27)	USA	Pigs (14)	Post-MI	Protons (30/40 Gy)	Scar homogenization (treated: 30.1% myocytes vs. untreated: 59.9%); 4 VT-related sudden deaths; stable cardiac function; MRI revealed dose-related tissue effects over time.
Takami et al (2021) (30)	Japan	Rabbits (32)	Whole LV Irradiation	Carbons + Protons (25 Gy)	Significant LV conduction delays (PR: PT25 > control, P = .003); reduced P and QRS voltages; sustained effects at 6 months; VF induced in 1 carbon beam rabbit; no VF in proton group; mild-moderate pericardial effusion in 19% (carbon) and 44% (proton) with no tamponade.
Vaskovskii et al (2022) (32)	Russia	Pigs (2)	AV Node & LV Ablation	Photons (40/45 Gy)	40 Gy induced transient AV block; 45 Gy resulted in permanent AV block and ventricular standstill by day 21; histology confirmed transmurality and precision.
Kim et al (2022) (29)	South Korea	Rats (9)	Proteomic (Healthy)	Photons (0/2/25 Gy)	25 Gy induced significant proteomic changes within 7 days; early effects on signal transduction, adhesion, and stress response; upregulation of oxidative stress proteins; potential mediators of early anti-arrhythmic effects identified.
Amino et al (2023) (22)	Japan	Rabbits (26)	HC, AT/AF & VT/VF	Carbons (15 Gy)	Radiation reduced AT/AF (1.2% vs. 9.9%) and VT/VF (1.2% vs. 7.8%); improved conduction velocity; reversed Cx40/43 downregulation and sympathetic nerve sprouting.
Imamura et al (2023) (31)	USA	Pigs (19)	Normal + Infarcted Myocardium	Protons (40 Gy)	Reduced bipolar voltage amplitude (normal: 10.1→5.7 mV, infarcted: 2.0→0.8 mV); conduction velocity decreased (normal: 85→55 cm/s, infarcted: 43.7→26.3 cm/s); Cx43 reduction observed from 1-week post-irradiation; myocytolysis, capillary hyperplasia, and dilation at 8 weeks.
Kancharla et al (2024) (28)	USA	Pigs (10)	Post-MI VA	Photons (25 Gy)	SBRT reduced VA inducibility (100% vs. 25%, P=0.07); scar density increased (33% vs. 14%, P=0.07); no fibrosis in remote myocardium; SBRT improved scar homogenization.

**Abbreviations:** AF: Atrial Fibrillation, AT: Atrial Tachycardia, AV: Atrioventricular, Cx40: Connexin-40, Cx43: Connexin-43, HC: Hypercholesterolemia, LVEF: Left Ventricular Ejection Fraction, MI: Myocardial Infarction, MRI: Magnetic Resonance Imaging, PET-CT: Positron Emission Tomography-Computed Tomography, PR: PR Interval, PT25: Proton Therapy 25 Gy, QRS: QRS Complex (ventricular depolarization), RMS40: Root Mean Square Voltage of the Last 40 ms, RSPV: Right Superior Pulmonary Vein, SBRT: Stereotactic Body Radiation Therapy, VT/VF: Ventricular Tachycardia/Fibrillation.

**Table 2.** Summary of Case Reports Investigating Stereotactic Arrhythmia Radioablation (STAR) for Ventricular Tachycardia (VT).

Author (Year)	Country	Age (Gender)	VT Type	Modality (Dose, fx)	Technology	PTV Volume (cc)	Motion Management
Loo et al (2015) (18)	USA	71 (M)	MMVT	Photons (25 Gy, 1fx)	CyberKnife	NR	Fiducial Marker
Jameau et al (2018) (33)	Switzerland	75 (M)	PMVT	Photons (25 Gy, 1fx)	CyberKnife	21	Fiducial Marker
Haskova et al (2018) (34, 35)	Czech Republic	34	PMVT	Photons (25 Gy, 1fx)	CyberKnife	62.2	NR
Bhaskaran et al (2019) (36)	Canada	34 (F)	MMVT	Photons (25 Gy, 1fx)	LINAC	52	4DCT, ITV
Zeng et al (2019) (37)	China	29 (M)	PMVT	Photons (25 Gy, 1fx)	CyberKnife	71.22	NR
Marti’-Almor et al (2020) (38)	Spain	64 (M)	MMVT	Photons (25 Gy, 1fx)	LINAC	NR	4DCT
Narducci et al (2020) (39)	Italy	60 (M)	MMVT	Photons (25 Gy, 1fx)	LINAC	303	4DCT, ITV
Mayinger et al (2020) (40)	Switzerland	71 (M)	MMVT	Photons (25 Gy, 1fx)	MRIdian	115.1	NR
Krug et al (2020) (41)	Germany	78 (M)	MMVT	Photons (25 Gy, 1fx)	LINAC	42.2	NR
Park and Choi (2020) (42)	South Korea	76 (M)	MMVT	Photons (25 Gy, 1fx)	LINAC	NR	NR
Dusi et al (2021) (43)	Italy	73 (M)	MMVT	Photons (25 Gy, 1fx)	NR	27.7	NR
Peichl et al (2021) (44) (35)	Czech Republic	66 (M)	MMVT	Photons (25 Gy, 1fx)	CyberKnife	18.3	NR
Amino et al (2021) (45)	Japan	75 (F)	PMVT	Photons (25 Gy, 1fx)	LINAC	49.7	NR
Quick et al (2021) (46)	Germany	85 (M)	MMVT	Photons (25 Gy, 1fx)	NR	8.51, 15.01	NR
Lee et al (2021) (47)	Korea	11 (M)	MMVT	Photons (25 Gy, 1fx)	LINAC	NR	4DCT, ITV
Kautzner et al (2021) (48)	Czech Republic	52 (M)	MMVT	Photons (25 Gy, 1fx)	LINAC	52	NR
		57 (M)	MMVT	Photons (25 Gy, 1fx)	LINAC	62.1	NR
		67 (M)	PMVT	Photons (25 Gy, 1fx)	LINAC	70	NR
Thosani et al (2021) (49)	USA	73 (M)	MMVT	Photons (25 Gy, 1fx)	LINAC	62.6	Margin
Aras et al (2021) (50)	Turkey	58 (M)	MMVT	Photons (25 Gy, 1fx)	LINAC	NR	4DCT, ITV
Li et al (2022) (51)	China	54 (M)	MMVT	Photons (25 Gy, 1fx)	LINAC	74.7	4DCT, ITV
Hayase et al (2022) (52)	USA	78 (M)	MMVT	Photons (25 Gy, 1fx)	LINAC	NR	NR
Levis et al (2022) (53)	Italy	73 (M)	MMVT	Photons (25 Gy, 1fx)	LINAC	89	4DCT
Haskova et al (2022) (35)	Czech Republic	77 (M)		Photons (25 Gy, 1fx)	CyberKnife	14.3	NR
Huang et al (2022) (54)	Taiwan	63 (M)	MMVT	Photons (12 Gy, 1fx)	LINAC	65.75	ITV
van der Ree et al (2022) (55)	Netherlands	60 (M)	PMVT	Photons (25 Gy, 1fx)	LINAC	300	4DCT, ITV
Wutzler et al (2022) (56)	Germany	56 (M)	PMVT	Photons (25 Gy, 1fx)	LINAC	NR	4DCT, ITV
Bernstein et al (2022) (57)	USA	75 (M)	MMVT	Photons (25 Gy, 1fx)	LINAC	87.9	4DCT
Kurzelowski et al (2022) (58)	Poland	69 (M)	MMVT	Photons (25 Gy, 1fx)	LINAC	56.37	DIBH
		72	MMVT	Photons (25 Gy, 1fx)	LINAC	56.72	DIBH
Cybulska et al (2022) (59)	Poland	67 (M)	PMVT	Photons (25 Gy, 1fx)	LINAC	NR	DIBH
Ninni et al (2022) (60)	France	42 (M)	MMVT	Photons (25 Gy, 1fx)	CyberKnife	NR	NR
Nasu et al (2022) (61)	Japan	58 (M)	PMVT	Photons (25 Gy, 1fx)	LINAC	29.1	4DCT
Pavone et al (2022) (62)	Italy	73 (M)	PMVT	Photons (25 Gy, 1fx)	LINAC	NR	4DCT, ITV
Cozzi et al (2022) (63)	Italy	81 (M)	MMVT	Photons (25 Gy, 1fx)	LINAC	122.5	4DCT, ITV
Mehrhof et al (2023) (64)	Germany	54 (M)	MMVT	Photons (25 Gy, 1fx)	CyberKnife	75.2	Fiducial Marker
		61 (M)	PMVT	Photons (25 Gy, 1fx)	LINAC	134.6	ITV
Jiwani et al (2023) (65)	USA	83 (M)	MMVT	Photons (25 Gy, 1fx)	LINAC	146.7	4DCT
van der Ree et al (2023) (66)	Netherlands	47 (F)	PMVT	Photons (2 Gy, 2fx; 20 Gy, 1fx)	CyberKnife	16	Fiducial Tracking
Kaestner et al (2023) (67)	Germany	63 (F)	MMVT	Photons (25 Gy, 1fx)	LINAC	NR	NR
Wijesuriya et al (2023) (68)	UK	69 (F)	MMVT	Photons (25 Gy, 1fx)	LINAC	NR	NR
Vaskovskii et al (2023) (69)	Russia	57 (M)	MMVT	Photons (25 Gy, 1fx)	LINAC	46	4DCT, ITV
Vozzolo et al (2023)	USA	44 (M)	MMVT	Photons (25 Gy, 1fx)	LINAC	NR	4DCT
Keyt et al (2023) (70)	USA	75 (M)	MMVT	Photons (25 Gy, 1fx)	LINAC	85	4DCT, ITV
Amino et al (2024) (71)	Japan	60 (M)	MMVT	Carbons (25 Gy, 1fx)	XiO (Elekta)	29.7	Motion Margin
Kautzner et al (2024) (72)	Czech Republic	54 (F)	MMVT	Photons (25 Gy, 1fx)	CyberKnife	NR	NR

**Abbreviations:** 4DCT: Four-Dimensional Computed Tomography, DIBH: Deep Inspiration Breath Hold, ITV: Internal Target Volume, LINAC: Linear Accelerator, MMVT: Monomorphic Ventricular Tachycardia, NR: Not Reported, PMVT: Polymorphic Ventricular Tachycardia.

**Table 3.** Summary of Clinical Trials and Case Series Investigating Stereotactic Arrhythmia Radioablation (STAR) for Ventricular Tachycardia (VT). If the entry is a clinical trial, its trial name is reported in the Author column.

Author (Year)	Country	Sample Size	Age (Median, Range)	Gender (M/F)	CM	LVEF(%) (Median, Range)	PTV Volume (cc)	Modality (Dose, fx)	Technology
Cuculich et al (2017) (19)	USA	5	62 (60–83)	4M/1F	2 ICM; 3 NICM	22 (15–26)	51.3 (17.3–81)	Photons (25 Gy, 1fx)	LINAC
Robinson et al (2019) (ENCORE-VT) (73)	USA	19	66 (49–81)	17M/2F	11 ICM; 8 NICM	25 (15–58)	98.9 (60.9–298.8)	Photons (25 Gy, 1fx)	LINAC
Chin et al (2020) (74)	USA	8	74 (65–86)	8M	4 ICM; 4 NICM	20 (15–32.5)	84.9 (21.1–190.7)	Photons (15–25 Gy, 1fx)	LINAC
Gianni et al (2020) (75)	USA	5	67 (45–76)	5M	4 ICM; 5 NICM	25 (20–55)	173 (80–184)	Photons (25 Gy, 1fx)	CyberKnife
Lee et al (2021) (76)	UK	7	70 (60–79)	4M/3F	5 ICM; 2 NICM	25 (15–45)	89.5 (57.5–139)	Photons (25 Gy, 1fx)	LINAC
Yugo et al (2021) (77)	Taiwan	3	68 (65–83)	2M/1F	3 NICM	44 (20–59)	70 (20–130)	Photons (25 Gy, 1fx)	LINAC
Ho et al (2021) (78)	USA	6	72.5 (64–77)	6M	2 ICM; 4 NICM	26 (10–46)	120.5 (66–193)	Photons (25 Gy, 1fx)	LINAC
Carbucicchio et al (2021) (STAR-MI-VT) (79)	Italy	7	72 (59–78)	7M	3 ICM; 4 NICM	21.1 (20.3–44.4)	198.3 (88.1–239)	Photons (25 Gy, 1fx)	LINAC
Qian et al (2022) (80)	USA	6	72 (70–73)	6M	6 ICM	20 (16–20)	319 (280–330)	Photons (25 Gy, 1fx)	LINAC
Wight et al (2022) (81, 82)	USA	14	60.5 (50–70)	10M/4F	5 ICM; 9 NICM	NR	NR	Photons (25 Gy, 1fx)	LINAC
Molon et al (2022) (83)	Italy	6	79.5 (61–85)	5M/1F	3 ICM; 3 NICM	26.5 (20–42)	NR	Photons (25 Gy, 1fx)	LINAC
Ninni et al (2022) (84)	France	17	68 (30–83)	13M/4F	10 ICM; 7 NICM	35 (20–53)	53.3 (19.96–185.88)	Photons (25 Gy, 1fx)	CyberKnife
Aras et al (2023) (85)	Turkey	8	61.5 (33–85)	8M	2 ICM; 6 NICM	25 (10–30)	157.4 (70.5–272.7)	Photons (25 Gy, 1fx)	LINAC
van der Ree et al (2023) STARNL-1 (86)	Netherlands	6	73 (54–83)	6M	6 ICM	38 (24–52)	187 (93–372)	Photons (25 Gy, 1fx)	LINAC
Krug et al (2023) RAVENTA (87)	Germany	5	67 (49–74)	4M/1F	2 ICM; 3 NICM	35 (20–45)	69.6 (43.4–80.7)	Photons (25 Gy, 1fx)	NR
Herrera Siklody et al (2023) (88)	Switzerland	20	68 (47–80)	15M/5F	6 ICM; 14 NICM	31 (20–72)	23 (14–115)	Photons (20–25 Gy, 1fx)	CyberKnife/MRIdian/LINAC
Miszczyk et al (2023) SMART-VT (89)	Czech Republic	11	67 (45–72)	10M/1F	9 ICM; 2 NICM	27 (20–40)	73 (18.6–111.3)	Photons (25 Gy, 1fx)	LINAC
Haskova et al (2024) (90)	Czech Republic	36	66 (56–76)	33M/3F	20 ICM; 16 NICM	31 (22,40)	39.4 (12.6–90.5)	Photons (25 Gy, 1fx)	CyberKnife
Arkles et al (2024) (91)	USA	15	65 (57.2–72.8)	13M/2F	7 ICM; 8 NICM	30.2 (26.6–33.8)	45.6 (84.7–124.1)	Photons (25 Gy, 1fx)	LINAC
Borzov et al (2024) (92)	Israel	3	64 (63–72)	3M	1 ICM; 2 NICM	27.5 (15–30)	49.7 (47.8–91.8)	Photons (25 Gy, 1fx)	LINAC
Bianchi et al (2024) (93)	Italy	11	68 (53–81)	11M	5 ICM; 6 NICM	40 (30–57)	90.4 (30.6119.5)	Photons (25 Gy, 1fx)	MRIdian

**Abbreviations:** CM: Cardiomyopathy, CyberKnife: Robotic Radiosurgery System, ICM: Ischemic Cardiomyopathy, LINAC: Linear Accelerator, LVEF: Left Ventricular Ejection Fraction, MRIdian: MRI-Guided Radiation Therapy System, NICM: Non-Ischemic Cardiomyopathy, NR: Not Reported, PTV: Planning Target Volume, fx: Fraction(s).

**Table 4.** Summary of Meta-Analysis Results for Stereotactic Arrhythmia Radioablation (STAR) in Ventricular Tachycardia (VT): Outcomes include pooled estimates for mortality at 6 and 12 months, reduction in VT burden at 6 months, and grade 3+ adverse events within 90 days. Subgroup analyses evaluate variations by treatment modality (LINAC vs. CyberKnife), LVEF (≤ median vs. > median), patient age (≤ median vs. > median), and cardiomyopathy type (ICM vs. NICM). Results are presented as pooled effect estimates with 95% confidence intervals (CI), Cochran’s Q statistics, and heterogeneity (I^2^).

Outcome	Metric	Overall	LINAC vs CyberKnife	LVEF (≤ Median vs > Median)	Age (≤ Median vs > Median)	Cardiomyopathy (ICM vs NICM)
Deaths at 6 months	Pooled Effect	0.16 (0.11, 0.21)	0.16 (0.11,0.22) vs 0.11 (0,0.23)	0.18 (0.09,0.26) vs 0.13 (0.06,0.19)	0.14 (0.09,0.19) vs 0.22 (0.11,0.33)	0.13 (0.06,0.20) vs 0.16 (0.09,0.23)
	Cochran's Q	16.95 (p=0.56)	14.68 (p=0.79) vs 0.07 (p=0.79)	4.51 (p=0.81) vs 8.58 (p=0.28)	10.72 (p=0.55) vs 4.45 (p=0.62)	2.09 (p=0.91) vs 11.53 (p=0.24)
	I^2^	0.00%	4.61% vs 0.00%	0.00% vs 18.41%	0.00% vs 0.00%	0.00% vs 21.94%
Deaths at 12 months	Pooled Effect	0.32 (0.26, 0.39)	0.35 (0.26,0.43) vs 0.33 (0.22,0.44)	0.33 (0.23,0.43) vs 0.31 (0.23,0.39)	0.31 (0.24,0.37) vs 0.41 (0.26,0.54)	0.33 (0.25,0.42) vs 0.30 (0.21,0.40)
	Cochran's Q	15.45 (p=0.56)	5.82 (p=0.88) vs 7.25 (p=0.03)	4.05 (p=0.85) vs 10.95 (p=0.09)	13.52 (p=0.26) vs 0.45 (p=0.99)	7.91 (p=0.34) vs 6.91 (p=0.44)
	I^2^	0.00%	0.00% vs 72.41%	0.00% vs 45.22%	18.64% vs 0.00%	11.48% vs 0.00%
Grade 3+ Adverse Events within 90 days	Pooled Effect	0.07 (0.04, 0.11)	0.08 (0.04,0.13) vs 0.06 (0.01,0.12)	0.10 (0.03,0.16) vs 0.06 (0.02,0.11)	0.07 (0.03,0.11) vs 0.08 (0.04,0.11)	0.10 (0.05,0.15) vs 0.05 (0.01,0.10)
	Cochran's Q	8.6 (p=0.99)	5.29 (p=097) vs 2.07 (p=0.55)	0.90 (p=0.99) vs 5.97 (p=0.65)	6.78 (p=0.91) vs 1.76 (p=0.91)	4.10 (p=0.90) vs 1.93 (p=0.90)
	I^2^	0.00%	0.00% vs 0.00%	0.00% vs 0.00%	0.00% vs 0.00%	0.00% vs 0.00%
VT events reduction at 6 months	Pooled Effect	0.75 (0.73, 0.77)	0.99 (0.97,1.00) vs 0.15 (0.02,0.28)	0.65 (0.60,0.69) vs 0.89 (0.79,0.99)	0.75 (0.72,0.79) vs 0.83 (0.72,0.95)	0.59 (0.54,0.64) vs 0.99 (0.97,1.01)
	Cochran's Q	1163.39 (p<0.05)	34.66 (p<0.05) vs 26.42 (p<0.05)	446.03 (p<0.05) vs 5.11 (p=0.40)	520.34 (p<0.05) vs 6.13 (p=0.29)	347.39 (p<0.05) vs 11.17 (p=0.08)
	I^2^	98.80%	71.15% vs 96.21%	98.65% vs 2.12%	98.85% vs 18.40%	98.56% vs 46.31%
