# Supplementary material for: Stereotactic Arrhythmia Radioablation for Refractory Ventricular Tachycardia: A Narrative Review and Exploratory Pooled Analysis of Clinical Outcomes and Toxicity
Source: ArXiv. 2025 May 14:arXiv:2501.18872v2. Originally published 2025 Jan 31. Preprint. [Version 2] (PMC11838787)
Supplement: 1 [file NIHPP2501.18872V2-supplement-1.pdf]

Supplementary Materials

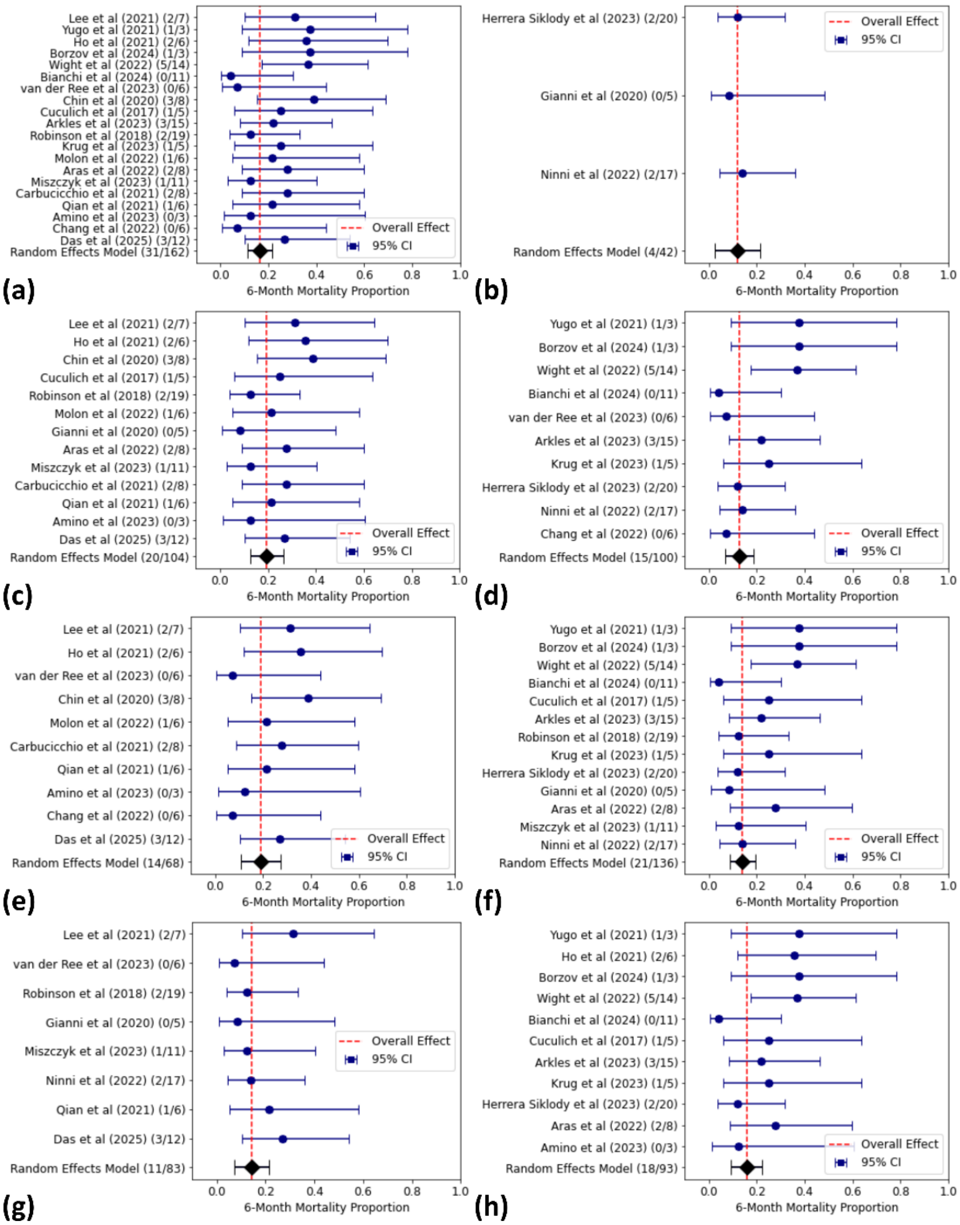

Figure S1. Forest plots depicting pooled mortality proportion 6 months following stereotactic arrhythmia radioablation (STAR), stratified by key subgroups. Each plot shows individual study estimates with 95% confidence intervals and an overall pooled estimate using a random-effects model. Studies are labeled with their reported VT reduction (%) and ordered chronologically. Subgroup comparisons include: **(A–B)** treatment modality — LINAC-based STAR **(A)** vs. CyberKnife **(B)**, **(C–D)** baseline left ventricular ejection fraction (LVEF) ≤ median **(C)** vs. > median **(D)**, **(E–F)** patient age ≤ median **(E)** vs. > median **(F)**, and **(G–H)** underlying cardiomyopathy — ischemic (ICM) **(G)** vs. non-ischemic (NICM) **(H)**.

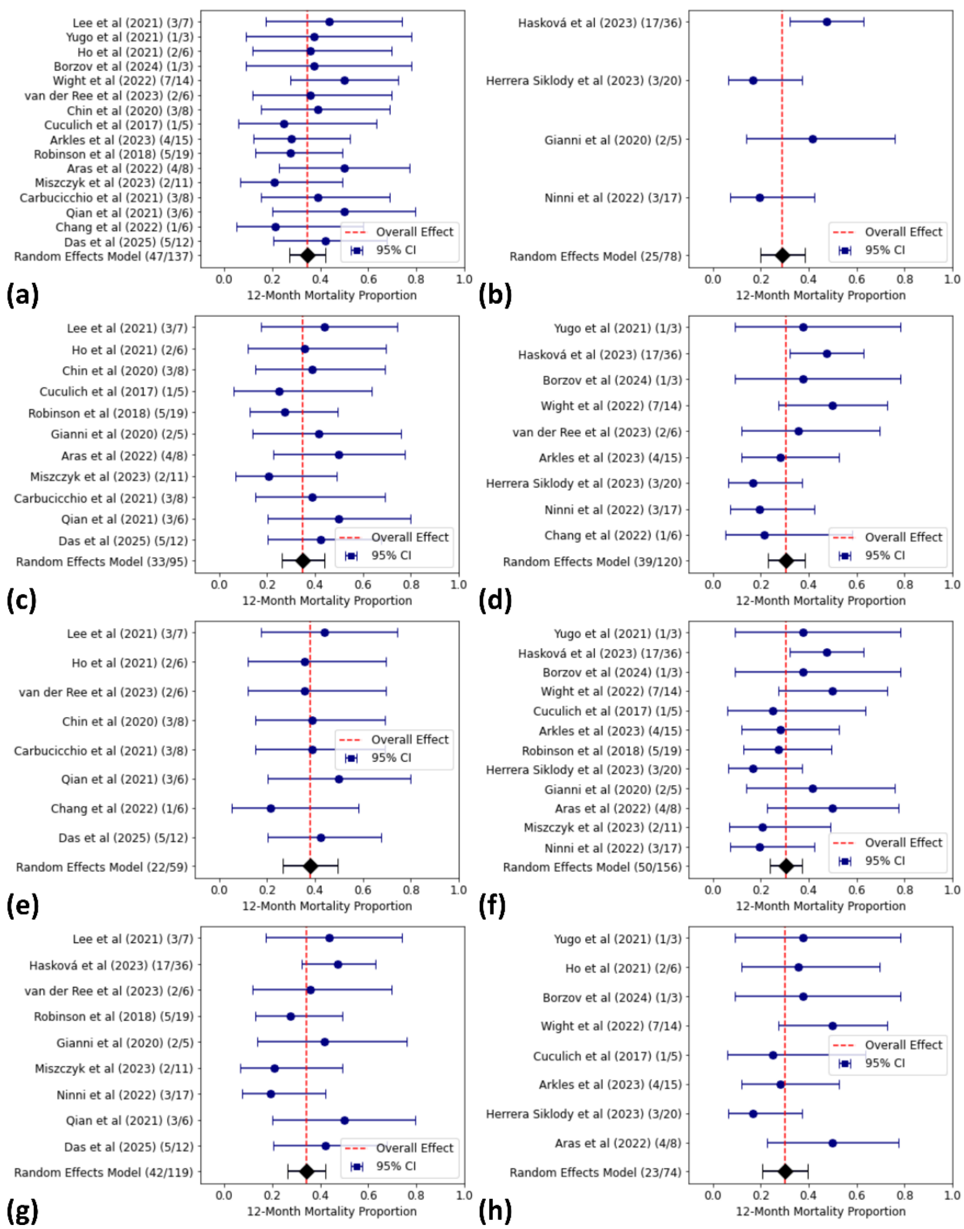

Figure S2. Forest plots depicting pooled mortality proportion 12 months following stereotactic arrhythmia radioablation (STAR), stratified by key subgroups. Each plot shows individual study estimates with 95% confidence intervals and an overall pooled estimate using a random-effects model. Studies are labeled with their reported VT reduction (%) and ordered chronologically. Subgroup comparisons include: **(A–B)** treatment modality — LINAC-based STAR **(A)** vs. CyberKnife **(B)**, **(C–D)** baseline left ventricular ejection fraction (LVEF) ≤ median **(C)** vs. > median **(D)**, **(E–F)** patient age ≤ median **(E)** vs. > median **(F)**, and **(G–H)** underlying cardiomyopathy — ischemic (ICM) **(G)** vs. non-ischemic (NICM) **(H)**.

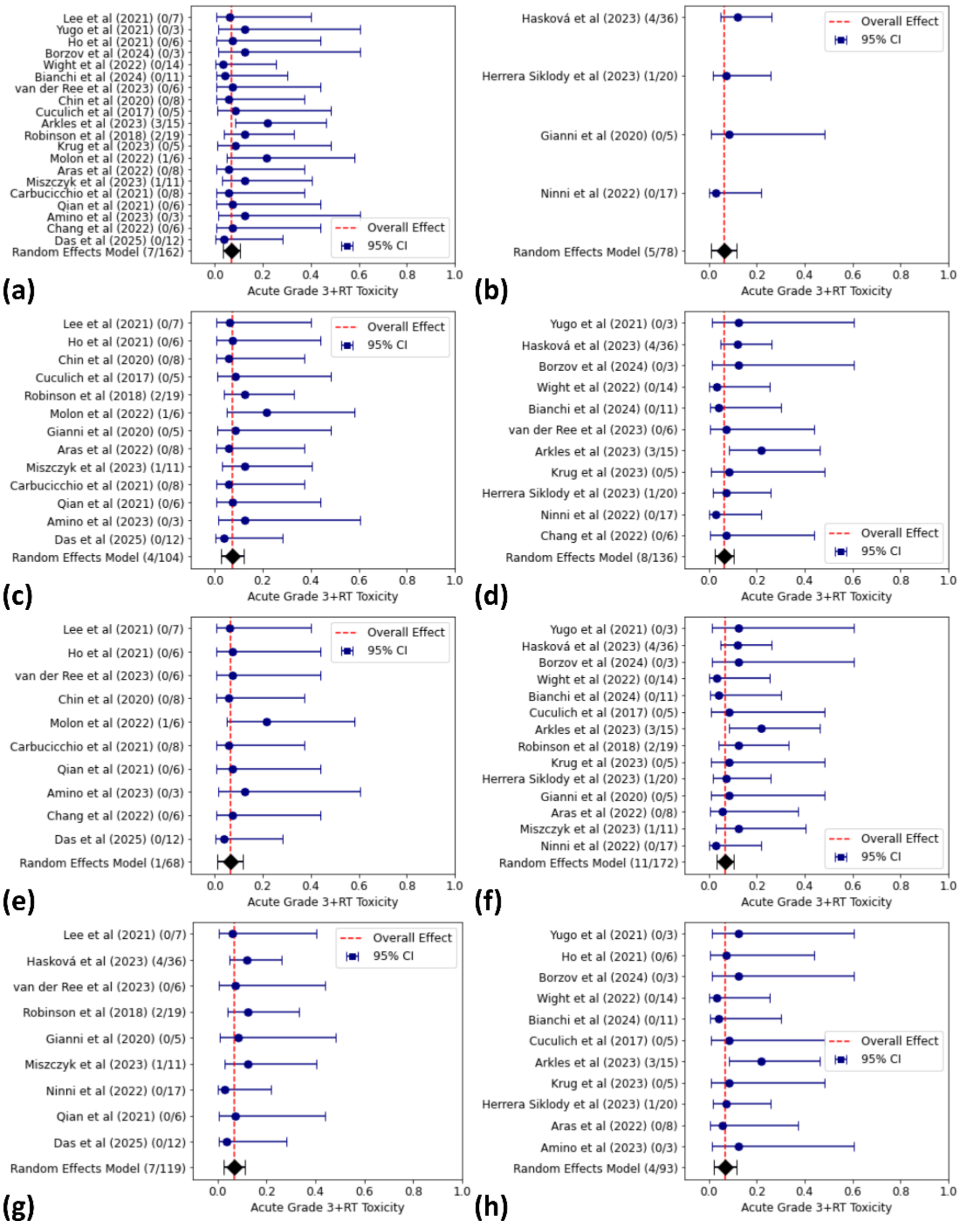

Figure S3. Forest plots depicting pooled acute adverse events following stereotactic arrhythmia radioablation (STAR), stratified by key subgroups. Each plot shows individual study estimates with 95% confidence intervals and an overall pooled estimate using a random-effects model. Studies are labeled with their reported VT reduction (%) and ordered chronologically. Subgroup comparisons include: **(A–B)** treatment modality — LINAC-based STAR **(A)** vs. CyberKnife **(B)**, **(C–D)** baseline left ventricular ejection fraction (LVEF) ≤ median **(C)** vs. > median **(D)**, **(E–F)** patient age ≤ median **(E)** vs. > median **(F)**, and **(G–H)** underlying cardiomyopathy — ischemic (ICM) **(G)** vs. non-ischemic (NICM) **(H)**.
